# A community health worker pilot intervention to increase patient portal use among people with HIV

**DOI:** 10.1371/journal.pdig.0001782

**Published:** 2026-10-29

**Authors:** Elena Whitney, Samantha A. Devlin, Eleanor E. Friedman, Joseph A. Mason, Sreevidhya Patil Vilas, Ainur Kagarmanova, Nehemiah Clark, Neda Laiteerapong, Jade Pagkas-Bather, Russell A. Brewer, John Schneider, Jon E. Grant, Harold Pollack, Daniela Bischoff, Jessica P. Ridgway

**Affiliations:** 1 Pritzker School of Medicine, The University of Chicago, Chicago, Illinois, United States of America; 2 Section of Infectious Diseases and Global Health, Department of Medicine, The University of Chicago, Chicago, Illinois, United States of America; 3 Section of General Internal Medicine, Department of Medicine, The University of Chicago, Chicago, Illinois, United States of America; 4 Department of Psychiatry and Behavioral Neuroscience, The University of Chicago, Chicago, Illinois, United States of America; 5 Crown Family School of Social Work, Policy, and Practice, The University of Chicago, Chicago, Illinois, United States of America; 6 Department of Public Health Sciences, The University of Chicago, Chicago, Illinois, United States of America; Instituto Politécnico Nacional Escuela Superior de Medicina: Instituto Politecnico Nacional Escuela Superior de Medicina, MEXICO

## Abstract

Use of patient portals is associated with improved engagement in care and health outcomes in a variety of healthcare contexts including among people with HIV (PWH). However, there are disparities in portal access and barriers to portal use including lack of awareness, need for more education, and mistrust or unfamiliarity with the technology used. We investigated whether a community health worker (CHW)-led pilot initiative could increase patient portal use among PWH. Participants who enrolled in the study received help with the patient portal (MyChart) and a one-time personalized 30-minute training session with the CHW on MyChart functions. Participants reported sociodemographic information, current MyChart use, barriers, and functions of interest. MyChart utilization data were retrieved for the six months before and after the training. Descriptive statistics were used to characterize the data, and McNemar’s exact tests were used to determine significant differences between pre-training and post-training intervals (a pre-post, single-arm study design). A total of 55 participants were enrolled and trained. The majority of participants were Black or African American (94.5%), and most had Medicaid or Medicare insurance (65.5%). The most frequently used MyChart functions included viewing lab results (65.5%) and health history (60.0%), scheduling appointments (56.4%), and sending messages (54.5%). The most commonly reported barriers were lack of time (21.8%) and not understanding how to use the portal (20.0%). There was a significant increase in read/view functions (reviewing test results, reading messages, etc.) after the training (61.8% to 81.8%, p-value = 0.048). The increase in use of write/update functions (responding to messages, paying bills, updating account information, etc.) was not statistically significant (56.4% to 72.7%, p-value = 0.216). The increase in use of some MyChart functions after the training indicates the potential of CHW-led interventions to address barriers and increase portal use among PWH.

## Author summary

Patient portals are secure websites that allow patients to access health information and communicate with their care team. Patient portals can help patients manage their healthcare, which is important for people with HIV (PWH). However, there are challenges in making sure everyone gets the chance to sign up and use a patient portal. We wanted to see if a training led by a community health worker (CHW) could increase patient portal use. For PWH who were interested, the CHW helped them enroll in the portal (if not already enrolled) and showed them how to use it. Participants also reported information about their current portal use and any issues they experienced with the portal. We documented portal use to view test results, view health history, schedule appointments, and message care team members. One of the most commonly reported challenges was not understanding how to use the portal. We found that the CHW training was successful in increasing use of portal functions such as viewing health information and updating the electronic medical record. Our study showed that a CHW-led training has the potential to increase some patient portal use among PWH.

## Introduction

Patient portals are secure websites or web-based applications that allow patients to access health information and communicate with providers [1]. Use of patient portals is associated with improved engagement in care and health outcomes for primary care and chronic diseases [1–5]. For people with HIV (PWH), the patient portal is a tool to facilitate HIV care and self-management, and there is high interest in using patient portals among PWH [6–8]. Additionally, studies have shown that portal use by PWH is associated with improved engagement in HIV care, including retention in care and adherence to antiretroviral therapy (ART), as well as improved HIV outcomes such as viral suppression [9–13]. Patient portals have also been shown to be useful as a population health approach to behavioral health screening [14].

Although patient portals have significant utility, interventions are needed to achieve equitable access and use. Studies have found disparities in portal access related to race, age, income, and education [15–18]. Among PWH specifically, Black patients are less likely to have portal access and to use portal functions including messaging, viewing lab results, and requesting prescription refills [9,19]. Structural factors that influence access to and use of patient portals among the general population include lack of access to internet and technology, issues with health literacy, and bias in offering patients access to the portal; these barriers also likely contribute to disparities in portal access and use among PWH [17,18,20,21]. Self-reported barriers to portal use among PWH have included lack of awareness, need for further education, and mistrust or unfamiliarity with the technology used, similar to the barriers reported by other populations [6,21–23]. Additionally, among PWH, concerns of stigmatization and loss of confidentiality are further barriers to portal use [24,25]. Strategies are needed to address these barriers and to better engage PWH in patient portal use. While previous studies have demonstrated the benefit of digital health interventions to promote engagement in HIV care, individualized educational interventions to increase use of existing patient portals have been less well studied among PWH [26–30].

One potential strategy to increase knowledge of and trust in patient portals is utilizing community health workers (CHWs). A CHW is a trained layperson who shares similar backgrounds and identities with the people they serve and correspondingly possesses a deep understanding of the community’s health and social needs. CHWs may promote equitable access by reaching underserved and vulnerable communities [31–34]. The CHW model specifically addresses the individual and interpersonal levels of influence in the digital environment within the Framework for Digital Health Equity. The Framework for Digital Health Equity [35] and the Technology Acceptance Model 2 (TAM2) [36] provide frameworks for interventions to increase access to and acceptance of technology, such as patient portals. The Framework for Digital Health Equity is an expansion of the National Institute on Minority Health Disparities (NIMHD) Research Framework [37], which measures determinants of health across various domains including biological, behavioral, physical/built environment, sociocultural environment, and the healthcare system, at different levels of the socio-economic model. The Framework for Digital Health Equity additionally incorporates digital determinants of health and includes these in the digital environment domain. Digital determinants include the following examples across the socio-economic model: individual (digital literacy, technology access, attitudes), interpersonal (implicit technology bias, shared devices), community (infrastructure, community technology norms), and societal (data standards, algorithmic biases, technology policy). TAM2 is a model for technology acceptance that explains how social influence and cognition impact the perceived usefulness and ease of using new technology and can ultimately lead to technology acceptance and technology use. This study aimed to increase patient portal use among PWH by utilizing a CHW embedded within an HIV care clinic to address patient barriers, guided by the Framework for Digital Health Equity, TAM2, as well as the Consolidated Framework for Research Implementation (CFIR) 2.0 [38].

## Materials and methods

### Ethics statement

This study was approved by the University of Chicago Institutional Review Board (IRB23–1687). Informed written consent was obtained from participants prior to enrollment in the study.

### Study design and setting

This study took place at the University of Chicago Medicine (UCM) Ryan White Adult HIV Care Clinic (AHCC), a provider of HIV prevention and care services for Chicago’s south side, a majority Black community disproportionately impacted by HIV.

### Intervention and data collection

The study team consisted of a CHW who was hired and trained to provide education and technical assistance for the existing patient portal used at UCM (MyChart), including information on enrollment and use of specific functions such as viewing medical information, (e.g., appointments, after-visit summaries, health histories, and lab results), sending messages, and responding to questionnaires. The CHW completed required Human Subjects Research training through the Collaborative Institutional Training Initiative (CITI) and received training on MyChart functions through training materials available on the Epic UserWeb [39]. The CHW also completed the New England Public Health Training Center Teach Back Training [40]; during this training, the CHW learned the teach back method, in which they asked patients to explain instructions using their own words to ensure clarity. The CHW performed mock training sessions with members of the research team prior to recruiting, consenting, and training participants.

The CHW recruited potential patients through MyChart, phone calls, and letters and in person during clinic visits to ask if they would like to sign up for MyChart and/or to learn more about it. PWH not enrolled in MyChart were prioritized for recruitment. Patients were eligible to participate if they were: (1) an adult (≥ 18 years old) patient with HIV; (2) received their HIV care at the UCM AHCC; (3) could speak and understand English; and (4) provided written informed consent. Exclusion criteria were individuals who had another person make their medical decisions or had visual and/or physical impairments that prevented them from using a computer or mobile device.

For PWH who consented to participate and did not have an active MyChart account, the CHW assisted them with enrolling in MyChart. For all participants (i.e., those with *and* without previously activated MyChart accounts), the CHW provided education on specific MyChart functions in a 30-minute in-person hands-on individual training. All participants received training in the basic functions of MyChart, privacy and confidentiality, and how to download the MyChart app and log into the interface. Depending on participants’ needs and preferences, additional training was provided regarding finding health information, scheduling appointments, viewing after-visit summaries, viewing laboratory results, viewing health history, sending messages to the care team, and filling out health questionnaires. The CHW maintained a checklist of training topics and completed it with participants to ensure intervention fidelity. After reviewing presentations and showing patients where to find additional training videos on MyChart, the CHW would note the training topics they covered with each patient and the patient’s perceived understanding of those topics.

Participants were surveyed regarding their current use of MyChart including what functions they currently use, any barriers to MyChart use, and what MyChart functions they were most interested in learning more about. Participants also reported their sociodemographic information including age, sex, race and ethnicity, sexual orientation, and education, as well as length of HIV diagnosis. Data were collected via REDCap [41]. The CHW followed up with each participant one month after the training intervention to answer questions and address any challenges. Data on participants’ MyChart activity were evaluated for the six months before and after training to determine if training increased MyChart use.

### Data analysis

Electronic health record (EHR) data regarding MyChart use were retrieved for six months prior to the date of training and six months after training to conduct a single-arm, pre-post study. The day of training was excluded from both the pre-training period and the post-training period. MyChart functions were described both as individual functions (viewing upcoming appointments, viewing health information, viewing financial information, viewing current medications, viewing messages from the care team, viewing health questionnaires, viewing test results, making appointments, updating financial information, writing messages to the care team, filling out health questionnaires, updating the medical record), as well as two aggregate categories: any read/view functions (e.g., viewing upcoming appointments, viewing financial information, viewing messages from the care team, etc.) and any write/update functions (e.g., making appointments, writing messages to the care team, updating account information, etc.). We used descriptive statistics to characterize the data into use of both individual MyChart functions and the aggregate categories of any read/view and any write/update actions. For categorical variables, we report frequencies, numbers, and percentages. For continuous variables, we present medians and interquartile (IQR) intervals. We used McNemar’s exact tests to determine significant differences in MyChart function use between pre-training and post-training six-month intervals. We corrected p-values to reflect multiple comparisons using the Holm-Bonferroni method [42], with a statistical significance level of ≤0.05.

## Results

A total of 55 participants were enrolled and trained in how to use the patient portal (MyChart) by the CHW. The median age was 46 years (IQR = 37–61); 67.3% were male; 94.5% were Black or African American; and 65.5% had Medicaid or Medicare insurance (Table 1). The majority of participants (98.2%) had already activated MyChart at the time of enrollment. Many participants (36.3%) reported that they used no MyChart functions frequently (Table 2). The MyChart functions participants reported using most frequently included viewing lab results (65.5%), viewing health history (60.0%), viewing appointments (56.4%), and sending messages to the care team (54.5%).

**Table 1:** Demographics of participants (N = 55).

Variable	Number (%)
Age (years) (median, IQR)	46 (37- 61)
Sex Female Male	18 (32.7%) 37 (67.3%)
Race Black or African-American Other/Unknown White	52 (94.5%) 1 (1.8%) 2 (3.6%)
Ethnicity Non-Hispanic Unknown	53 (96.4%) 2 (3.6%)
Insurer Medicaid Medicare Private	26 (47.3%) 10 (18.2%) 19 (34.5%)
Duration of HIV infection <1 year 1–9 10–19 20–29 30–39 Unknown	2 (3.6%) 8 (14.5%) 5 (9.1%) 3 (5.4%) 7 (12.7%) 30 (54.5%)
Sexual Orientation Gay/Lesbian Heterosexual/Straight Other/Unknown	17 (30.9%) 16 (29.1%) 22 (40%)
Education More than college College graduate Some college or technical school High school graduate Some high school	3 (5.5%) 5 (9.1%) 17 (30.9%) 27 (49.1%) 3 (5.5%)
MyChart activation status Activated Pending Activation	54 (98.2%) 1 (1.8%)

**Table 2:** MyChart functions used by participants, self-reported by participants prior to training (N = 55).

MyChart function	Number (%)
Viewing scheduled appointments	31 (56.4%)
Scheduling appointments	21 (38.2%)
Viewing after-visit summaries	31 (56.4%)
Viewing lab results	36 (65.5%)
Viewing my health history	33 (60.0%)
Sending messages to my care team	30 (54.5%)
Filling out questionnaires about my health	6 (10.9%)
Other	0 (0.0%)
None	19 (34.5%)

The most commonly reported barriers to MyChart use were lack of time (21.8%), not understanding how to use MyChart (20.0%), forgotten password (16.4%), and forgotten username (10.9%) (Table 3). Finally, we asked what MyChart functions participants were most interested in learning more about. Frequently reported functions included filling out health questionnaires (56.4%), viewing lab results (43.6%), health history (43.6%), and after-visit summaries (41.8%) and sending messages to the care team (40.0%) (Table 4).

**Table 3:** Patient reported barriers to MyChart use (N = 55).

Variable	Number (%)
Concerns about confidentiality	2 (3.6%)
No time	12 (21.8%)
Do not understand how to use	11 (20.0%)
Forgot password	9 (16.4%)
Forgot username	6 (10.9%)
Forget that I can use MyChart	3 (5.5%)
Unable to log in to email	3 (5.5%)
No access to internet	1 (1.8%)

**Table 4:** MyChart features patients wished to learn more about, self-reported by participants prior to training (N = 55).

MyChart function	Number (%)
Logging in	12 (21.8%)
Retrieving password	12 (21.8%)
Viewing after-visit summaries	22 (40.0%)
Viewing lab results	24 (43.6%)
Viewing my health history	24 (43.6%)
Sending messages to my care team	22 (40.0%)
Filling out questionnaires about my health	31 (56.4%)
Other	0 (0.0%)
None	5 (9.1%)

In the six months prior to training, 61.8% of participants had viewed and 56.4% had updated MyChart. When we compared MyChart functions from the six months prior to training to the six months after training, use of all functions increased, except viewing billing information. Overall read/view functions increased significantly in the post-training period (from 61.8% to 81.8%, p-value = 0.048) (Table 5). Viewing any part of MyChart significantly increased after the intervention (61.8% to 81.8%, p-value = 0.048), as well as viewing questionnaires (21.8% to 43.6%, p-value = 0.050). However, viewing laboratory test results did not significantly increase (45.5% to 65.5%, p-value = 0.074). Some write/update functions did not increase significantly in the six-month period after training compared to the six-month period before training, including making appointments (23.6% to 41.8%, p-value = 0.216), filling out health questionnaires (21.8% to 40.0%, p-value = 0.116), and any write/update function (56.4% to 72.7%, p-value = 0.216). Updating the medical record (43.6% to 65.5%, p-value = 0.050) significantly increased after the training.

**Table 5:** MyChart functions used in the six months prior to training and six months after training (N = 55).

MyChart function	Total	Six months prior Number (%)	Six months after Number (%)	P-value
View any part of MyChart Yes No	55 (100.0%) 0 (0.0%)	34 (61.8%) 21 (38.2%)	45 (81.8%) 10 (18.2%)	0.048
View upcoming appointments Yes No	54 (98.2%) 1 (1.8%)	33 (60.0%) 22 (40.0%)	42 (76.4%) 13 (23.6%)	0.180
View health information Yes No	55 (100.0%) 0 (0.0%)	34 (61.8%) 21 (38.2%)	45 (81.8%) 10 (18.2%)	0.048
View financial information Yes No	32 (58.2%) 23 (41.8%)	13 (23.6%) 42 (76.4%)	13 (23.6%) 42 (76.4%)	0.999
View current medications Yes No	45 (81.8%) 10 (18.2%)	20 (36.4%) 35 (63.6%)	27 (49.1%) 28 (50.9%)	0.369
View messages from care team Yes No	45 (81.8%) 10 (18.2%)	26 (47.3%) 29 (52.7%)	34 (61.8%) 21 (38.2%)	0.216
View health questionnaires Yes No	39 (70.9%) 16 (29.1%)	12 (21.8%) 43 (78.2%)	24 (43.6%) 31 (56.4%)	0.050
View test results Yes No	48 (87.3%) 7 (12.7%)	25 (45.5%) 30 (54.5%)	36 (65.5%) 19 (34.5%)	0.074
Any function requiring writing/updating Yes No	54 (98.2%) 1 (1.8%)	31 (56.4%) 24 (43.6%)	40 (72.7%) 15 (27.3%)	0.216
Make appointments Yes No	36 (65.5%) 19 (34.5%)	13 (23.6%) 42 (76.4%)	23 (41.8%) 32 (58.2%)	0.216
Updating finances/billing Yes No	35 (63.6%) 20 (36.4%)	12 (21.8%) 43 (78.2%)	17 (30.9%) 38 (69.1%)	0.665
Writing messages to care team Yes No	39 (70.9%) 16 (29.1%)	18 (32.7%) 37 (67.3%)	24 (43.6%) 31 (56.4%)	0.369
Filling out health questionnaires Yes No	37 (67.3%) 18 (32.7%)	12 (21.8%) 43 (78.2%)	22 (40.0%) 33 (60.0%)	0.116
Updating the medical record Yes No	51 (92.7%) 4 (7.3%)	24 (43.6%) 31 (56.4%)	36 (65.5%) 19 (34.5%)	0.050

## Discussion

Our study found an increase in several MyChart functions after a CHW-led training, demonstrating the potential of CHW-based interventions to increase patient portal use among PWH, particularly among those already enrolled in the portal. While the training led to increased use of MyChart to view information, it was less effective in promoting use of the platform for entering information, perhaps due to less participant-reported interest in some of these functions. Overall, however, our results are consistent with literature supporting the usefulness of individual-level interventions to increase portal use in general populations [29,30].

The intervention tested in this study involved a combination of enrollment assistance and individualized education on how to use the patient portal. This is similar to previous studies across inpatient and outpatient settings that aimed to increase patient portal use, most of which involved education-based interventions [29,30]. In our study, we saw a high level of enrollment in the patient portal, which resulted in the CHW spending more time on portal functions and use rather than enrollment and initial introduction to the portal. Several studies have utilized patient navigators to assist with portal enrollment and use, while others used a variety of education modalities including videos, group training, and individualized training [29,43–45]*.* Our intervention utilized a CHW who did not have a preexisting support relationship with patients, but was able to conduct outreach beyond clinic visits, potentially increasing the reach of the intervention. Non-education interventions that have been studied include email reminders, providing tablets to patients, and system-level interventions such as universal access [11,46,47]. Most studies showed increased portal use after the intervention, measured by portal enrollment or logins.

Of the few studies that have evaluated specific portal functions following interventions, viewing lab results and messaging care teams were the most commonly increased portal functions [43,45]. Neither of these functions significantly increased after the intervention in our study. However, viewing any part of the patient portal, viewing health information, and viewing health questionnaires significantly increased after the training, as well as updating the medical record. PWH were not represented in these prior studies of specific portal functions and may have specific barriers to portal use and desires for portal functionality that were addressed by the CHW as needed [24,25,48].

We found that PWH frequently used MyChart to view health information and health questionnaires, similar to patterns of patient portal use described among PWH and in other clinical contexts [11,19,49,50]. Increasing use of these and other MyChart functions may improve engagement in care [4,11,51]. Among PWH specifically, viewing information on the patient portal has been associated with improved ART adherence and viral suppression [11]. While we found increased use of several portal functions following the CHW-led training, additional studies are needed to further determine the most effective initiative for improving overall use of patient portals among PWH.

One of the most significant barriers to portal use in our study population was not understanding how to use the portal, which is likely explained by factors such as age and/or digital health familiarity. The CHW intervention directly targeted this barrier by providing individualized education. At the onset of the study, most PWH were registered for MyChart, but roughly one third did not actively use the portal. Similar barriers to portal use beyond registration have also been demonstrated in previous studies [6,19]. The increased use of specific portal functions we observed following the training suggests that individualized education may be a way to address these barriers.

There are known disparities in portal access across age, race, ethnicity, and socioeconomic status [15,16,18]. Universal portal registration is a strategy that has previously been shown to mitigate disparities in portal access [47]. The majority of participants in this study were Black or African American, a group that is usually less likely to have access to patient portals [15,18]. However, our study saw high levels of patient portal enrollment at baseline, making it difficult to assess disparities in access. Increased portal use following the intervention in this study points to the potential of a CHW-led intervention to address racial disparities in portal use, but this will have to be tested in a larger, non-pilot study.

This study has several limitations to acknowledge. The study was conducted at a single site at an urban academic center among a small population of PWH, which may limit generalizability to other settings. The intervention was implemented by a single CHW, which may limit reproducibility. Relatedly, our CHW was hired specifically for this research project. However, health systems could use existing CHWs for a similar initiative, which could promote sustainability of such an intervention. This study had a small sample size, which may have limited the ability to observe significant changes in portal function use after the intervention. As a single-arm, pre-post pilot study, there are limitations in how strongly we can assert that the training was responsible for the observed increases in MyChart use. In this study, we evaluated portal use via a binary variable (use or not use). Other means of examining increases in portal use, including the frequency of use of each function as well as aggregate functions, might provide a more in-depth examination of portal use before and after the intervention. Finally, selection bias might have occurred in which PWH who are more actively engaged in the healthcare system were more likely to participate, indicated by the fact that most study participants had active MyChart accounts prior to enrollment and may represent a group with increased interest in using the patient portal. The tested intervention may be less effective among patients with limited interest in portal use or who face technological, accessibility, or language barriers.

The increase in use of serval MyChart functions following the training in this study supports the potential of CHW-led interventions to increase patient portal use among PWH, particularly those who already have access to and familiarity with the portal. CHWs are uniquely positioned to build trust, provide culturally and linguistically concordant support, and address barriers to portal use among PWH. As they are embedded within the communities they serve, CHWs offer a promising person-centered strategy for expanding patient portal access and meaningful use [52]. This study only examined the impact of the intervention on portal use, so future directions should include an evaluation of the impact on HIV care continuum outcomes.

## References

[1] Carini E, Villani L, Pezzullo AM, Gentili A, Barbara A, Ricciardi W, et al. The impact of digital patient portals on health outcomes, system efficiency, and patient attitudes: updated systematic literature review. J Med Internet Res. 2021;23(9):e26189. doi: 10.2196/26189 34494966 PMC8459217

[2] Brands MR, Gouw SC, Beestrum M, Cronin RM, Fijnvandraat K, Badawy SM. Patient-centered digital health records and their effects on health outcomes: Systematic review. J Med Internet Res. 2022;24(12):e43086. doi: 10.2196/43086 36548034 PMC9816956

[3] Anderi E, Eisman EB, Bryant A, Hunt T, Mahal M, Vaughn I, et al. Patient portal frequency of use in patients with hypertension and diabetes. J Am Board Fam Med. 2025;38(5):868–76. doi: 10.3122/jabfm.2025.250069R1 41491560 PMC12978604

[4] Silver RA, Subramaniam C, Stylianou A. Association between patient portal use and office visits. J Med Syst. 2021;45(7):74. doi: 10.1007/s10916-021-01749-0 34097149

[5] Graetz I, Huang J, Muelly ER, Fireman B, Hsu J, Reed ME. Association of mobile patient portal access with diabetes medication adherence and glycemic levels among adults with diabetes. JAMA Netw Open. 2020;3(2):e1921429. doi: 10.1001/jamanetworkopen.2019.21429 32074289 PMC7646995

[6] Chu D, Schuster T, Lessard D, Mate K, Engler K, Ma Y, et al. Acceptability of a Patient Portal (Opal) in HIV clinical care: A feasibility study. J Pers Med. 2021;11(2):134. doi: 10.3390/jpm11020134 33669439 PMC7920437

[7] Hall A, Devlin S, Won J, Schmitt J, J PR. Electronic patient portal use among people living with HIV. J Med Internet Res. 2023;25:e47740. 10.2196/47740 PMC10698658 37988161

[8] Luque AE, van Keken A, Winters P, Keefer MC, Sanders M, Fiscella K. Barriers and facilitators of online patient portals to personal health records among persons living with HIV: Formative research. JMIR Res Protoc. 2013;2(1):e8. doi: 10.2196/resprot.2302 23612564 PMC3628162

[9] Schember CO, Scott SE, Jenkins CA, Rebeiro PF, Turner M, Furukawa SS, et al. Electronic patient portal access, retention in care, and viral suppression among people living with HIV in Southeastern United States: Observational study. JMIR Med Inform. 2022;10(7):e34712. doi: 10.2196/34712 35877160 PMC9361138

[10] Saberi P, Catz SL, Leyden WA, Stewart C, Ralston JD, Horberg MA. Antiretroviral therapy adherence and use of an electronic shared medical record among people living with HIV. AIDS Behav. 2015;19(Suppl 2):177–85. 10.1007/s10461-014-0982-x PMC4497945 25572829

[11] Midboe AM, Cave S, Shimada SL, Griffin AC, Avoundjian T, Asch SM, et al. Relationship between patient portal tool use and medication adherence and viral load among patients living with HIV. J Gen Intern Med. 2024;39(Suppl 1):127–35. doi: 10.1007/s11606-023-08474-z 38252241 PMC10937883

[12] Ridgway JP, Zimmer D, Shetty R, Laiteerapong N. Association between electronic patient portal enrollment and HIV care outcomes among people living with HIV at an urban academic medical center. AIDS Behav. 2026;30(1):315–8. doi: 10.1007/s10461-025-04869-7 40880006 PMC12443438

[13] McInnes DK, Shimada SL, Midboe AM, Nazi KM, Zhao S, Wu J, et al. Patient use of electronic prescription refill and secure messaging and its association with undetectable HIV viral load: A retrospective cohort study. J Med Internet Res. 2017;19(2):e34. doi: 10.2196/jmir.6932 28202428 PMC5332835

[14] Franco MI, Staab EM, Zhu M, Knitter A, Wan W, Gibbons R, et al. Pragmatic clinical trial of population health, portal-based depression screening: the PORTAL-depression study. J Gen Intern Med. 2023;38(4):857–64. doi: 10.1007/s11606-022-07779-9 36127535 PMC9488885

[15] Sinha S, Garriga M, Naik N, McSteen BW, Odisho AY, Lin A, et al. Disparities in electronic health record patient portal enrollment among oncology patients. JAMA Oncol. 2021;7(6):935–7. doi: 10.1001/jamaoncol.2021.0540 33830178 PMC8033503

[16] Khatib R, Glowacki N, Chang E, Lauffenburger J, Pletcher MJ, Siddiqi A. Disparities in patient portal engagement among patients with hypertension treated in primary care. JAMA Netw Open. 2024;7(5):e2411649. doi: 10.1001/jamanetworkopen.2024.11649 38748420 PMC11096988

[17] Richwine C, Johnson C, Patel V. Disparities in patient portal access and the role of providers in encouraging access and use. J Am Med Inform Assoc. 2023;30(2):308–17. doi: 10.1093/jamia/ocac227 36451262 PMC9846679

[18] Yoon E, Hur S, Opsasnick L, Huang W, Batio S, Curtis LM, et al. Disparities in patient portal use among adults with chronic conditions. JAMA Netw Open. 2024;7(2):e240680. doi: 10.1001/jamanetworkopen.2024.0680 38421645 PMC10905301

[19] Avoundjian T, Troszak L, Cave S, Shimada S, McInnes K, Midboe AM. Correlates of personal health record registration and utilization among veterans with HIV. JAMIA Open. 2021;4(2):ooab029. doi: 10.1093/jamiaopen/ooab029 34278241 PMC8280933

[20] Chehab M, Engler K, Schuster T, Lessard D, Osmanlliu E, Cadri A, et al. Studied factors and metrics of patient portal use among people with HIV: A rapid scoping review. AIDS Care. 2025;37(8):1366–79. doi: 10.1080/09540121.2025.2532596 40705755

[21] Maini I, Gilotra K, Sadigh G. Patient factors associated with the use of online portal health information in the postpandemic era: Cross-sectional analysis of a national survey. J Med Internet Res. 2025;27:e60472. doi: 10.2196/60472 40179386 PMC12006766

[22] Conti J, Fix GM, Javier SJ, Cheng H, Perez T, Dunlap S, et al. Patient and provider perspectives of personal health record use: A multisite qualitative study in HIV care settings. Transl Behav Med. 2023;13(7):475–85. doi: 10.1093/tbm/ibac118 37084300

[23] Turner K, Clary A, Hong YR, Alishahi Tabriz A, Shea CM. Patient portal barriers and group differences: Cross-sectional national survey study. J Med Internet Res. 2020;22(9):e18870. 10.2196/18870 PMC7530687 32940620

[24] Chu D, Lessard D, Laymouna MA, Engler K, Schuster T, Ma Y, et al. Understanding the risks and benefits of a patient portal configured for HIV care: Patient and healthcare professional perspectives. J Pers Med. 2022;12(2):314. doi: 10.3390/jpm12020314 35207803 PMC8880024

[25] Walker JA, Staab EM, Ridgway JP, Schmitt J, Franco MI, Hunter S, et al. Patient perspectives on portal-based anxiety and depression screening in HIV care: A qualitative study using the consolidated framework for implementation research. Int J Environ Res Public Health. 2024;21(6):692. doi: 10.3390/ijerph21060692 38928937 PMC11203430

[26] Carroll JK, Tobin JN, Luque A, Farah S, Sanders M, Cassells A, et al. “Get Ready and Empowered About Treatment” (GREAT) study: A pragmatic randomized controlled trial of activation in persons living with HIV. J Gen Intern Med. 2019;34(9):1782–9. doi: 10.1007/s11606-019-05102-7 31240605 PMC6712153

[27] Kim GS, Kim L, Baek S, Kwon S, Kim JM, Choi JY, et al. Providing 2 Types of mHealth interventions to support self-management among people living with HIV: Randomized clinical trial. JMIR Mhealth Uhealth. 2025;13:e60905. doi: 10.2196/60905 40444823 PMC12140505

[28] Schnall R, Sanabria G, Jia H, Cho H, Bushover B, Reynolds NR, et al. Efficacy of an mHealth self-management intervention for persons living with HIV: The WiseApp randomized clinical trial. J Am Med Inform Assoc. 2023;30(3):418–26. doi: 10.1093/jamia/ocac233 36469808 PMC9933073

[29] Lyles CR, Nelson EC, Frampton S, Dykes PC, Cemballi AG, Sarkar U. Using electronic health record portals to improve patient engagement: Research priorities and best practices. Ann Intern Med. 2020;172(11 Suppl):S123–9. doi: 10.7326/M19-0876 32479176 PMC7800164

[30] Grossman LV, Masterson Creber RM, Benda NC, Wright D, Vawdrey DK, Ancker JS. Interventions to increase patient portal use in vulnerable populations: A systematic review. J Am Med Inform Assoc. 2019;26(8–9):855–70. doi: 10.1093/jamia/ocz023 30958532 PMC6696508

[31] Scott K, Beckham SW, Gross M, Pariyo G, Rao KD, Cometto G, et al. What do we know about community-based health worker programs? A systematic review of existing reviews on community health workers. Hum Resour Health. 2018;16(1):39. doi: 10.1186/s12960-018-0304-x 30115074 PMC6097220

[32] Kenya S, Jones J, Arheart K, Kobetz E, Chida N, Baer S, et al. Using community health workers to improve clinical outcomes among people living with HIV: A randomized controlled trial. AIDS Behav. 2013;17(9):2927–34. doi: 10.1007/s10461-013-0440-1 23515640 PMC4184095

[33] Doblecki-Lewis S, Butts S, Botero V, Klose K, Cardenas G, Feaster D. A randomized study of passive versus active PrEP patient navigation for a heterogeneous population at risk for HIV in South Florida. J Int Assoc Provid AIDS Care. 2019;18:2325958219848848. doi: 10.1177/2325958219848848 31131679 PMC6748484

[34] Drainoni M-L, Baughman AL, Bachman SS, Bowers-Sword R, Davoust M, Fortu K, et al. Integrating community health workers into HIV care teams: Impact on HIV care outcomes. J HIV/AIDS Soc Serv. 2020;19(3):204–19. doi: 10.1080/15381501.2020.1785364

[35] Richardson S, Lawrence K, Schoenthaler AM, Mann D. A framework for digital health equity. NPJ Digit Med. 2022;5(1):119. doi: 10.1038/s41746-022-00663-0 35982146 PMC9387425

[36] Venkatesh V, Davis FD. A Theoretical extension of the technology acceptance model: four longitudinal field studies. Manag Sci. 2000;46(2):186–204. doi: 10.1287/mnsc.46.2.186.11926

[37] National Institute on Minority Health and Health Disparities. NIMHD research framework details. 2017. https://www.nimhd.nih.gov/resources/research-framework/nimhd-research-framework-details

[38] Damschroder LJ, Reardon CM, Opra Widerquist MA, Lowery J. Conceptualizing outcomes for use with the Consolidated Framework for Implementation Research (CFIR): The CFIR outcomes addendum. Implement Sci. 2022;17(1):7. doi: 10.1186/s13012-021-01181-5 35065675 PMC8783408

[39] Epic Systems Corporation. Epic UserWeb.

[40] Community health workers learn teach back. https://www.nephtc.org/enrol/index.php?id=70

[41] Harris PA, Taylor R, Thielke R, Payne J, Gonzalez N, Conde JG. Research electronic data capture (REDCap)--a metadata-driven methodology and workflow process for providing translational research informatics support. J Biomed Inform. 2009;42(2):377–81. doi: 10.1016/j.jbi.2008.08.010 18929686 PMC2700030

[42] Holm S. A simple sequentially rejective multiple test procedure. Scandinav J Stat. 1979;6:65–70.

[43] Matthews AK, Steffen AD, Burke LA, Donenberg G, Duangchan C, Akufo J, et al. The use of navigators to increase patient portal enrollment among patients in a federally qualified health care system. Ethn Dis. 2024;DECIPHeR(Spec Issue):117–25. doi: 10.18865/ed.DECIPHeR.117 38846728 PMC11895548

[44] Rodriguez JA, Zelen M, Szulak J, Moore K, Park L. A system-wide approach to digital equity: The digital access coordinator program in primary care. J Am Med Inform Assoc. 2024;31(7):1583–7. doi: 10.1093/jamia/ocae104 38741288 PMC11187422

[45] Weisner CM, Chi FW, Lu Y, Ross TB, Wood SB, Hinman A, et al. Examination of the effects of an intervention aiming to link patients receiving addiction treatment with health care: The LINKAGE clinical trial. JAMA Psych. 2016;73(8):804–14. doi: 10.1001/jamapsychiatry.2016.0970 27332703 PMC4972645

[46] Fujioka JK, Bickford J, Gritke J, Stamenova V, Jamieson T, Bhatia RS, et al. Implementation strategies to improve engagement with a multi-institutional patient portal: Multimethod study. J Med Internet Res. 2021;23(10):e28924. doi: 10.2196/28924 34709195 PMC8587179

[47] Milanfar L, Soulsby WD, Ling N, O’Brien JS, Oates A, McCulloch CE. Automatic enrollment in patient portal systems mitigates the digital divide in healthcare: An interrupted time series analysis of an autoenrollment workflow intervention. J Med Syst. 2024;48(1):94. doi: 10.1007/s10916-024-02114-7 39377862 PMC11461562

[48] Jackman K-M, Agwu A, Ohene-Kyei ET, Barfield A, Huettner S, Trent M. Perceptions about using a patient portal to self-disclose HIV-related information to sexual partners: A qualitative study among adolescents and emerging adults living with HIV. AIDS Care. 2025;37(12):2215–28. doi: 10.1080/09540121.2025.2562233 41289150 PMC12883172

[49] Steitz BD, Turer RW, Lin C-T, MacDonald S, Salmi L, Wright A, et al. Perspectives of patients about immediate access to test results through an online patient portal. JAMA Netw Open. 2023;6(3):e233572. doi: 10.1001/jamanetworkopen.2023.3572 36939703 PMC10028486

[50] Scheckel B, Schmidt K, Stock S, Redaèlli M. Patient portals as facilitators of engagement in patients with diabetes and chronic heart disease: scoping review of usage and usability. J Med Internet Res. 2023;25:e38447. doi: 10.2196/38447 37624629 PMC10492174

[51] Sukhija-Cohen AC, Patani H, Blasingame MF, Vu KL, Bastani R. Retention in HIV primary care using a web-based patient engagement platform: Multistate case-control study. J Med Internet Res. 2024;26:e64092. doi: 10.2196/64092 39357049 PMC11483251

[52] Kim K, Choi JS, Choi E, Nieman CL, Joo JH, Lin FR, et al. Effects of community-based health worker interventions to improve chronic disease management and care among vulnerable populations: A systematic review. Am J Public Health. 2016;106(4):e3–28. doi: 10.2105/AJPH.2015.302987 26890177 PMC4785041

